# Bacterial cellulose: Enhancing productivity and material properties through repeated harvest

**DOI:** 10.1016/j.bioflm.2025.100276

**Published:** 2025-03-26

**Authors:** N. Rackov, N. Janßen, A. Akkache, B. Drotleff, B. Beyer, E. Scoppola, N.E. Vrana, R. Hengge, C.M. Bidan, S. Hathroubi

**Affiliations:** a Institüt für Biologie/Mikrobiologie, Humboldt-Universität zu Berlin, 10555, Berlin, Germany; b Max Planck Institute of Colloids and Interfaces, Department of Biomaterials, 14476, Potsdam, Germany; c SPARTHA Medical, CRBS, 1 Rue Eugène Boeckel, 67000, Strasbourg, France; d European Molecular Biology Laboratory (EMBL), 69117, Heidelberg, Germany; e Cluster of Excellence, Matters of Activity, Sophienstrasse 22A, 10178, Berlin, Germany

**Keywords:** Bacterial cellulose, Intermittent harvesting, Adaptation, Mutation, Materials properties, Stress response

## Abstract

Bacterial cellulose (BC), a promising versatile biopolymer produced by bacteria, has an immense potential in various industries. However, large-scale application is hindered by high production costs and low yields. This study introduces an innovative approach combining a prolonged static culturing with intermittent harvesting. This novel strategy resulted in a significant increase in BC productivity, achieving up to a threefold rise in biomass within the first 35 days. Prolonged growth and continuous harvesting not only enhanced productivity but also led to a mutant strain M2 with higher yields and distinct BC architecture. Mechanical and structural analyses revealed that sequential harvest correlated with increasing crystallinity, altered crystallite sizes, and improved stiffness of the dry material during initial cycles, potentially reflecting bacteria adaptation to resources limitations. Genomic analysis identified key mutations in the M2 strain, including one in the RelA/SpoT enzyme, suggesting a reduced stringent response that promotes growth under nutrient-limiting conditions. Untargeted metabolomic profiling revealed deregulation of several metabolites, including a significant difference in fatty acid metabolites that could potentially influence membrane fluidity and BC secretion. Such metabolic and structural adaptations enhance BC production efficiency and material properties. These findings highlight the potential of intermittent harvesting for sustainable BC production and the role of bacterial adaptation in tuning BC properties. Further research will optimize this strategy and expand its applications in developing tailored biomaterials for diverse industries.

## 1 Introduction

Bacterial cellulose (BC) is a natural biopolymer produced by a wide variety of bacteria as a fibrous structural component in the extracellular matrix of biofilms. Unlike plant cellulose, which contains lignin, hemicellulose, pectin, and other biogenic components, BC purity is very high. BC thus requires minimal purification, which substantially reduces environmental pollution associated with the disposal of industrial waste during alkaline extraction and peroxide bleaching [1]. Although its molecular structure is similar to plant cellulose, BC is characterized by higher crystallinity (70–80 %), higher water holding capacity (up to 99 %), higher porosity, strong biocompatibility, and it outperforms plant-based cellulose in several mechanical properties, e.g., tensile properties and stiffness [[2], [3], [4]].

Many bacteria produce cellulose, such as *Escherichia coli, Salmonella enterica*, the plant pathogen *Dickeya dadantii*, soil bacteria *Burkholderia* *spp.* And *Pseudomonas putida* as well as several members of the *Komagataeibacter* and *Gluconacetobacter* genera, which are used as models for studying cellulose biosynthesis processes [5,6]. *Gluconacetobacter hansenii* ATCC 53582 used in this work, is one the highest bacterial cellulose producer reported and has been used as a valuable model aiming to increase cellulose productivity [7]. During fermentation of carbohydrates by *G. hansenii*, BC is formed as a gel-like, cellulose-based translucent pellicle mass on the surface of the culture fluid. Cellulose production is regulated by the presence of oxygen and the carbon source. When glucose is the main carbon source, the synthesis of cellulose occurs through the four enzymatic steps: Glucose → glucose-6-phosphate → glucose-1-phosphate → UDP-glucose → Cellulose [5].

BC-forming bacteria can be cultured in static or agitated conditions. In agitated culture, cell growth is promoted, glucose conversion occurs rapidly, leading to the formation of cellulose as pellets or clumps [8]. However, agitation also increases shear stress, which favors the selection of cellulose non-producing cells, which in turn drastically reduces cellulose yield [[9], [10], [11], [12]]. For BC production, static cultivation is preferred, as it minimizes shear stress, reduces the emergence of cellulose-deficient mutants, and - despite a lower initial production rate is more productive in the long term [12]. In static conditions, the cellulose pellicle is thought to retain aerobic bacterial cells at the air-liquid interface where the oxygen level is high [8,13]. Over days, BC gradually covers the whole interface, and new layers are added on the surface, increasing the thickness of the pellicle [14,15]. However, the production stops when the pellicle reaches a certain thickness due to the restricted access to nutrients by the active cellulose producer cells located at the top layers [16]. In addition, reduced oxygenation, depletion of the carbon source, accumulation of toxic metabolites and acidification of the medium result in inactivation of the bacteria in the pellicle (after around 8–20 days depending on the strain and the growth conditions) [17]. Alternative methods were explored, such as the use of bioreactor to optimize BC production [18,19]. Hsieh et al. [15] used a static cultivation technique with intermittent feeding, where they added fresh media onto the formed cellulose pellicle [15]. The active cells at the top layers of cellulose were continuously fed and the productivity was significantly enhanced. This study showed that cellulose layers were stacked up to become almost one single piece of BC, however, the first layers remained in the media and were exposed to evolutive conditions, i.e., acidification of media and depletion in glucose.

To overcome these current limitations in BC production, a creative approach inspired by a previous interdisciplinary and artistic work [20] was proposed and developed in the present study. To test how a removal of the cellulose pellicle along with the active cells located in the top layers [21] influences the rest of the culture, static growth has been combined with intermittent harvest of BC produced by *G. hansenii.* BC productivity as well as the properties of BC produced and harvested over time were screened by weighing and using mechanical tests, X-ray diffraction and electron microscopy. The isolation and characterization of a naturally selected mutant that shows higher cellulose productivity and exhibits unique properties is also reported. Untargeted metabolomic profiling was employed to reveal the metabolite landscape of both wildtype (WT) strain and the high-cellulose producer mutant referred to as strain M2.

## 2 Results

### 2.1 Bacterial cellulose production under intermittent harvests

**Fig. 1:**
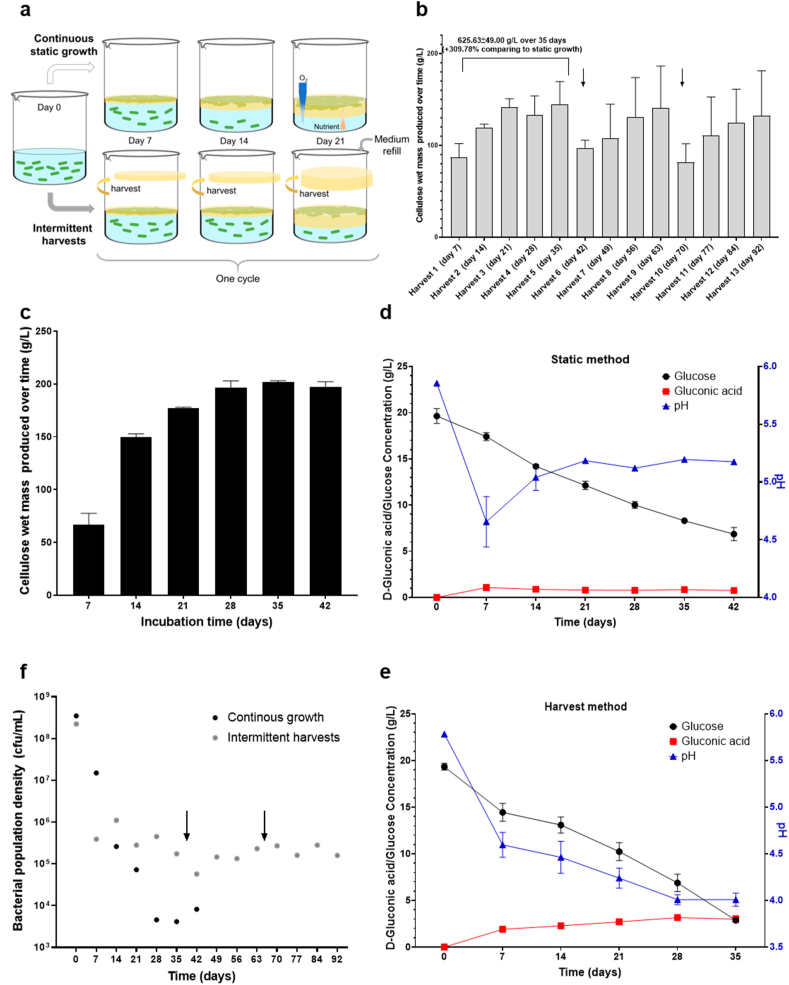
**Bacterial cellulose intermittent harvests vs continuous static growth.** (a) Sketch of the two different BC growth strategies. (b) Production of BC wet mass per liter of culture with the intermittent harvests, N = 6 and (c) during continuous static growth, N = 6. (d) Glucose consumption and gluconic acid production during continuous BC growth, N = 3. (e) Glucose consumption and gluconic acid production during BC growth with intermittent harvests, N = 3. (f) Evaluation of bacterial growth by CFU counting during BC growth, average of N = 2. Arrows represent the day when media was refilled with fresh HS media to the starting volume.

A primary objective of this study was to enhance the production of bacterial cellulose (BC) by *G. hansenii*. Initially, BC production was assessed under static conditions over various incubation periods. Notably, a BC pellicle materialized within 2–3 days of growth at the air-liquid interface, with BC cellulose layers progressively thickening with time. To improve BC productivity, our approach involved cultivating *G. hansenii* under static conditions while implementing a weekly cellulose harvest strategy (Fig. 1a). Cellulose productivity was meticulously monitored on a weekly basis, extending our observation period up to 92 days of growth, and conducted in-depth characterizations of the accumulated cellulose layers over time. After 7 days of incubation, the BC wet mass reached a range of 70–80 g/L of culture. After removing the cellulose layer, another BC layer was formed during the following 7 days and reached ∼120 g/L. Similarly, the next 3 weeks also showed a production of BC with yields even higher than during the first two weeks (around 130–150 g/L for each harvest) (Fig. 1b). When culture was left to grow for 21 or 28 days in the absence of weekly harvest, the biomass yield in BC reached approximately 150 g/L wet weight at day 21 and about 177 g/L by day 28. The highest wet mass was observed for pellicles grown for 35 days, reaching ∼196 g/L of wet weight, equivalent to ∼5.6 g/L of dry weight (Fig. 1c).

In contrast, the harvesting method yielded a total of 625.63 ± 49.00 g/L of wet mass BC and 8.17 ± 0.46 g/L of dry mass BC over the first 35 days. This represents a remarkable increase in productivity, approximatively 309 % for wet mass BC and 139 % for dry mass BC when compared to the static growth method (Fig. 1b and c). Furthermore, BC productivity using harvesting method showed significant improvement, surging from 12.41 g/L/day, which closely mirrors rates achieved during static growth over the same period, to levels reaching up to 19 g/L/day when *G. hansenii* was cultured for either 28 or 35 days.

The cellulose yield demonstrated a concomitant increase in tandem with the utilization of glucose throughout the cultivation period. Glucose was continuously consumed throughout the cultivation process. Under static condition, 50 % of the initial 20 g/L glucose was depleted around the day 28 with a relatively stable rate of glucose consumption, to a final value of around 9 g/L at day 35 (Fig. 1d). In contrast, with the application of the harvesting method, the glucose concentration dipped below 5 g/L by day 35, i.e. nearly half of what remained in the static growth condition at the same time point (Fig. 1e).

A significant by-product of glucose metabolism is gluconic acid, which arises from the oxidation of glucose. This not only depletes glucose, reducing its availability for BC production, but also lowers the pH, which further inhibits the biosynthesis of BC. In static experiments, gluconic acid reached ∼1 g/L by day 7, dropping the pH to ∼4.6. After 7 days, gluconic acid stabilized around 0.8 g/L, while pH rose to 5.1 by day 21. When harvested, gluconic acid levels peaked around 3 g/L by day 35, explaining the lower pH of ∼4 (Fig. 1d and e).

It is crucial to highlight each harvesting event resulted in a loss of media due to the water holding capacity of BC. Consequently, it was imperative to replenish the culture media after the fifth and ninth harvests (Fig. 1b and Fig. S1a; arrows at day 35 and day 63). This replenishment process led to an increased concentration of glucose and initiated another cycle of BC harvest, which was followed by glucose consumption to reach a concentration of 3 g/L after 4 weeks (four harvests). Interestingly, BC productivity decreases after each refill and falls at an average of around 11 and 12 g/L/day but then increases again to 18–20 g/L/day after four weeks (Fig. S1a). This initial drop is likely caused by the stress from sudden pH change and a surge in glucose concentration when fresh medium is added (Fig. S1a). However, after an adaptation period, *G. hansenii* adjusts to these conditions, resulting in the observed recovery in productivity (Fig. 1b and).

The bacterial growth in the liquid medium was determined by CFU counting (Fig. 1f). The starting inoculum contained 10^8^ CFU/mL cells to ensure the same number of cells in each beaker. As expected, bacteria rapidly join the neo-formed BC at the air-liquid interface. In continuous growth conditions, the concentration of planktonic, viable and culturable cells in the media decreased over time until day 28 (3.5 × 10^8^ CFU/mL to 4.6 × 10^3^), then, the planktonic population stayed constant around 10^3^ CFU/mL until day 42 (Fig. 1f). Several factors could explain this decrease in CFU count, including the accumulation of metabolic byproducts, limited oxygen availability and cells adhesion to the pellicle, which progressively reduces planktonic cells population over time. Harvesting had a clear impact on the concentration of viable and culturable cells, which in the first cycle decreased only to 10^5^ CFU/mL after 21 days, suggesting that harvesting likely helped maintain planktonic cell viability, probably by frequently exposing them to oxygen (Fig. 1f). The concentration of living cells in the liquid medium was then stable over the cycles until the end of the incubation.

We also examined the morphology of the colonies [22] isolated during the CFU counting. The ratio of cellulose-producing cells or Cel^+^ (producing rugous, convex microcolonies) to cellulose non-producing cells or Cel^−^ (producing smooth and flat microcolonies) was also noted and compared over incubation times by counting both types of morphology observed on Hestrin-Schramm (HS) agar plates. Although the initial planktonic inoculum contained some Cel^−^ cells (around 35 %), the Cel^+^ colonies dominated and even increased overtime to represent up to 99.5 % of the total population in the liquid medium in both growth conditions. No differences have been observed due the intermittent harvesting (Fig. S2). In conclusion, implementing a weekly cellulose harvest strategy significantly increased BC production.

### 2.2 Bacterial cellulose mechanical and structural properties over harvests

BC is known for its high mechanical strength which is much higher than commonly used biopolymers including plant cellulose [4,23]. Since culturing condition is an essential parameter for the BC structural and physical properties, the harvested cellulose was characterized as a function of harvest time, at the macroscopic and microscopic levels [14].

**Fig. 2:**
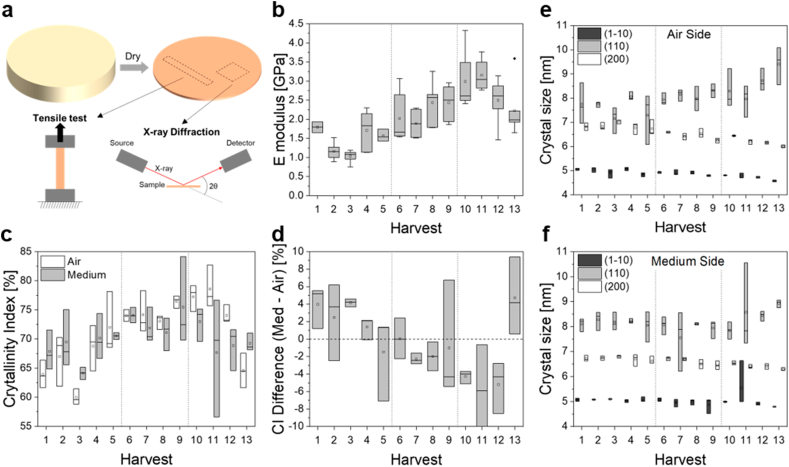
**Mechanical and structural properties of the BC harvested every week.** (a) Sketch showing BC sampling from thermally dried samples for tensile tests and XRD experiments. (b) E-modulus determined from tensile experiments performed on thermally dried biofilms produced during intermittent harvesting experiments. N = 3 to 10 per harvest. (c) Crystallinity index (CI) determined from XRD measurements performed on both the medium and air sides of BC samples produced during the intermittent harvesting experiments. (d) CI difference between the medium and the air sides of the BC samples. (e, f) BC crystal dimensions in directions orthogonal to their three main cellulose lattice planes determined from XRD measurements (1–10, 110 and 200) on both the medium (e) and the air side (f). Dotted vertical lines represent HS medium refill to the starting volume.

To test how BC mechanical properties were affected by the harvest method, tensile tests were applied on thermally dried BC samples collected during the different harvest cycles (Fig. 2a). The E-moduli that represent the resistance of materials to elastic deformations, were determined from the stress-strain curves obtained on each sample (Fig. 2b). The E-modulus of the BC collected during the first six harvests was found to lay between 1 and 2 GPa. However, this value then increased to reach a maximum of 3.4 GPa at the 11th harvest. Finally, the E-modulus of the materials collected during the last two harvests appeared to drop sharply.

The origin of these changes in the nanostructure of the material were then studied. For this, the crystallinity index (CI) of the oven-dried samples from the different harvests were estimated by performing XRD measurements on both sides of the samples to assess potential effects of the different growth conditions since cells and nutrient supply come from the medium while oxygen supply comes from the air (a representative diffractogram and its analysis is shown in Fig. S3). The evolution of the CI of the BC on the air side appeared to follow a similar trend as the E-modulus throughout the intermittent harvesting experiment. Indeed, the CI on the air side increased until the 11th harvest from roughly 64 %–78 % before decreasing drastically for the last two biofilm harvests (Fig. 2c). This trend was much less visible on the CI measured on the medium side, which only showed some fluctuation between 67 and 74 % (Fig. 2c). Interestingly, calculating the CI difference between the two sample sides revealed that early harvests consistently have a higher CI on the medium side compared to the air side until the 5th harvests (Fig. 2d). After this point, the trend is reversed and the CI becomes higher on the air side until the 12th and last harvest, where the CI is higher on the medium side again. It is noteworthy that the addition of fresh medium did not seem to influence the CI of the BC. Three distinctive peaks of the same XRD diffractograms were used to estimate the dimensions of the BC crystallites along each of these three crystalline directions (Fig. 2e and f). Harvest after harvest, the crystallites tend to become smaller in the directions orthogonal to the 1–10 and the 200 lattice planes. However, the third dimension of the crystallites (110) remains constant until the 11th harvest, from which it increases rapidly until the end of the experiment. No major difference in crystallite dimensions was observed between the two sides of the BC film. Note that due to instrumental particularities, only relative differences between CI and between crystal dimensions were highlighted but not conclusions were drawn on the absolute values.

### 2.3 Emergence of variants showing higher cellulose production

**Fig. 3:**
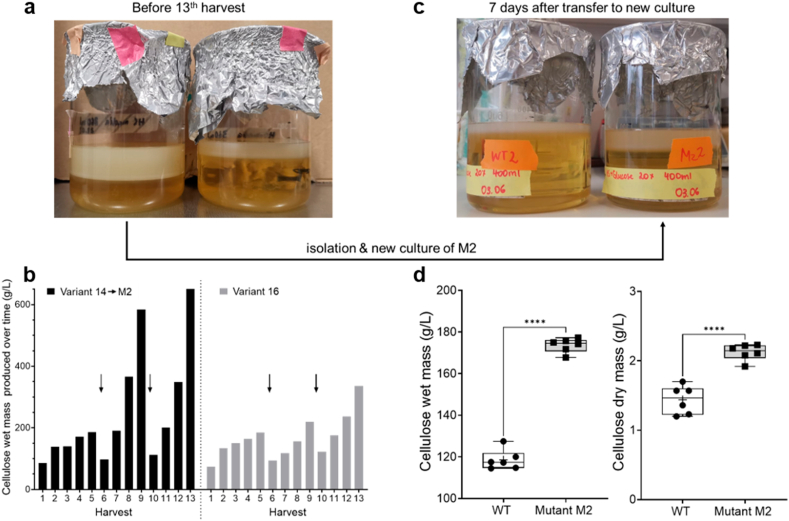
**Emergence of two variants with higher BC masses and isolation of a mutant with increased BC productivity.** (a) Example of a culture yielding thicker BC films (right) compared to average (left). (b) BC production by two variants. Arrows represent the day when media was refilled with fresh HS media to the starting volume. The most productive variant 14 was called mutant 2 (M2) and isolated to start a new culture. (c) Comparison between wild type (WT) and mutant 2 (M2) wet and dry BC masses after 7 days incubations. Three biological replicates and two technical duplicates were used for this study. ∗∗∗∗, *P* < 0,0001.

Repeating the harvests was shown to lead not only to a continuously increased production of cellulose within 7 days, but also to the emergence of several variants showing thicker BC (Fig. 3a). One culture showed a pronounced increase of cellulose yield at day 56 (8th harvest) and reached a maximum of 690 g/L (wet mass) produced within one week (Fig. 3b). The bacterial population were thus screened for mutants yielding thicker cellulose layers, which allowed us to isolate and purify a colony that was called strain M2 and compared it to the wild type (WT) strain. Fig. 3c shows that after 7 days of growth, M2 produced more wet cellulose (+1.4-fold) and also dry cellulose (+1.2-fold) compared to WT.

### 2.4 Structural and mechanical properties of BC from mutant M2 differ from those of the WT

**Fig. 4:**
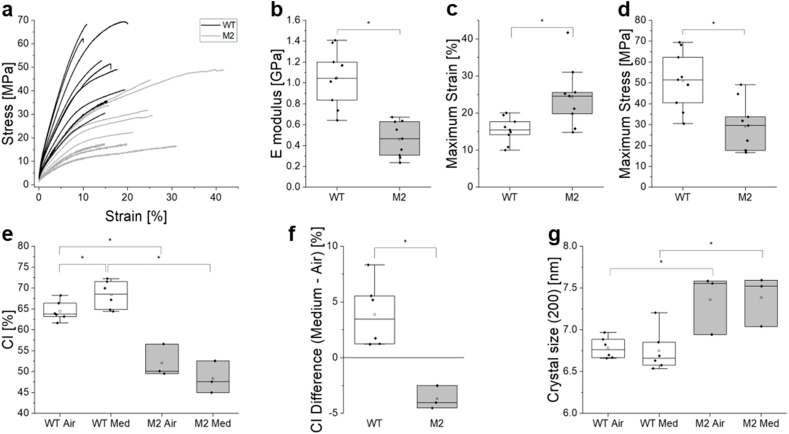
**Materials properties of BC from WT and M2 bacteria.** (a) Stress – strain curves obtained from tensile tests performed on thermally dried BC samples obtained after 7 days of growth from WT and M2 bacteria. (b) E-moduli, (c) maximum strain and (d) maximum stress determined from (a). (e) Crystallinity index (CI) determined from XRD measurements performed on both the medium and air sides of BC samples obtained after 7 days of growth from WT and M2 bacteria. (f) CI difference between the medium and the air sides of the BC samples. (g) BC crystal size determined from XRD measurements in the direction orthogonal to the cellulose lattice plane (200) on both the medium and the air sides.

To verify if growing bacterial cellulose with the mutant M2 also leads to changes in BC materials properties, mechanical and structural investigations were performed on samples obtained from the WT and the M2 mutant grown simultaneously under the same conditions. Therefore, cellulose grown for 7 days from both the WT and the M2 mutant was thermally dried and subjected to tensile experiments to estimate their respective mechanical properties. The resulting stress-strain curves revealed that dried BC from the M2 mutant was more compliant to deformation than dried BC from the WT (Fig. 4a) and their corresponding E-moduli differed by more than a factor 2 (0.45 GPa for M2 versus 1 GPa for WT) (Fig. 4b). Further analyses showed that while dried BC from M2 culture was more deformable (Fig. 4c), dried BC from WT was twofold stronger (Fig. 4d). The toughness of the two materials was not significantly different though (Fig. S4).

Accordingly, the values of crystallinity index (CI) obtained on the BC produced by WT bacteria were significantly higher (around 65 %) compared to M2 BC (around 50 %) (Fig. 4e). However, while higher CI were measured on the medium side of the BC harvested from WT cultures compared to their side exposed to the air, the reversed trend was observed on BC samples produced by the M2 mutants. This phenomenon is highlighted by calculating the CI difference between the medium and the air side of each individual sample (Fig. 4f). On both sides of the samples, BC crystals appeared to be slightly larger in the direction orthogonal to the (200) plane in samples obtained from M2 compared to WT cultures (Fig. 4g).

### 2.5 Bacterial cellulose from the WT and mutant M2 have different structural profiles

To complement the structural comparison of the BC obtained from the WT and M2 strains, the morphology along cross-sections of lyophilized samples was observed using environmental scanning electron microscopy (ESEM) and crystallography profiles were measured on oven-dried BC cross-sections using a synchrotron X-ray source. Note that while lyophilization allows to keep the structure at the mesoscopic scale, the strong dehydration may modify the properties of the crystals, hence the choice of different drying methods.

**Fig. 5:**
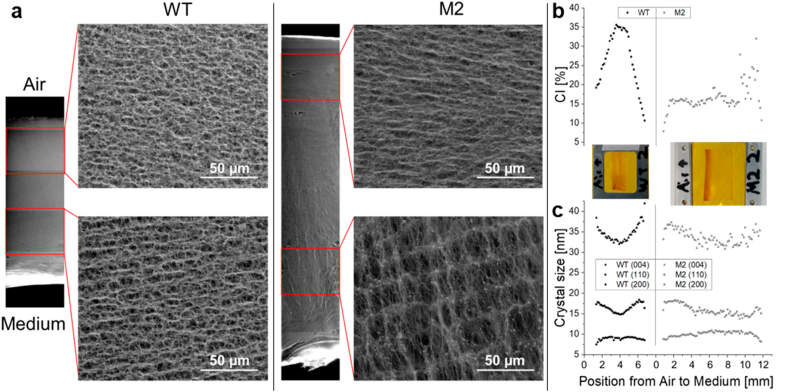
**Structural profiles of BC from WT and M2.** (a) ESEM images of WT and M2 lyophilized biofilm cross sections after 7 days of growth. Note the larger inter-layer distance in the lower half of the M2 mutant biofilm compared to the WT biofilm. (b) Profile of BC crystallinity index and (c) profile of crystal size along different lattice directions, both derived from X ray scattering data obtained across the dry cross sections of WT and M2 pellicles displayed.

The ESEM images revealed distinct organizations in the mutant M2 and the WT (Fig. 5a). The upper half of the biofilms, which is on the air side, was similar in both the M2 and the WT samples, consisting of a mesh of cellulose fibers with pores of μm size. Notably, the fibers seemed to have a more pronounced orientation along the interface in the BC produced by the mutant. However, the lower half of the M2 biofilms, which is on the medium side, consisted of fibrous layers with inter-layer distances of 10–15 μm, while it consisted of a more random micrometric porous mesh in the WT biofilm (Fig. 5a).

Series of X-ray diffractograms were acquired every 150 μm along a straight line from the air side to the medium side on dried BC cross-sections from M2 and WT cultures. The diffractograms obtained on these samples revealed notable differences compared to the ones obtained on the air and medium sides of dried BC (Fig. S5). Indeed, five peaks could be identified, corresponding to the cellulose lattice planes (1-10), (110), (102), (200) and (004) (Table S1). Only the more prominent peaks were considered to compute the crystallinity indices (CI) and the crystal size was only computed along the (110) and the (200) directions. Fig. 5b shows that the crystallinity of the BC was not homogeneous across the biofilm, with the central part of the film showing a large part of crystalline phase in the case of the WT. In contrast, the BC sample obtained from the mutant M2 seemed to have a lower but more homogenous crystallinity, except close to the medium side, where it was slightly higher. Overall, the CI of the BC produced by the WT was higher compared to the M2 (Fig. S6a), as already observed from the measurements on the dried biofilms (Fig. 4e). However, it is interesting to note that the CI profile measured on the dried cross-sections of the WT sample pointed toward a higher crystallinity on the air side, whereas the XRD measurements performed on both surfaces of the dried biofilms indicated the opposite (Fig. 4e). This opposite trend is also true for the BC samples harvested from M2 and could be attributed to the different geometries of the two experimental setups. In the XRD laboratory device, the incident X-ray source and the detector are positioned on the same side of the BC sample, whereas at the synchrotron, the X-ray goes through the sample and the detector is on the other side. When measuring anisotropic materials such as the BC samples (Fig. 5a), such techniques are thus sensitive to the orientation of the samples, and this is to be considered in the interpretation of the results.

The three more prominent peaks of the diffractograms were used to estimate the dimensions of the BC crystals in the direction perpendicular to the corresponding lattice planes, i.e. (110), (200) and (004). In the BC sample harvested from the WT culture, the variations of crystal dimensions either followed the trends observed for the CI (e.g., the crystal size perpendicular to the (200) plane was larger in the middle of the cross-section than at the interfaces) or this trend was mirrored as observed for the crystal dimensions perpendicular to the planes (110) and (004) (Fig. 5c). While a similar observation could be made on the BC sample from the M2 mutant, the sharp increase of CI observed close to the interface with the medium was not much reflected in the crystal size distribution (Fig. 5c). Compared to the BC crystals produced by the WT, the crystals produced by the mutant M2 seemed to be larger in the direction perpendicular to the (200) plane and smaller in the direction perpendicular to the (110) plane (Fig. S6b). Although significant, this last difference was not detected in the XRD measurements performed with the laboratory XRD device on the dried biofilms (Fig. S7).

Taken together, the M2 mutant not only showed an increased yield of cellulose production, but also changes in structure, morphology, and mechanical properties of the BC produced.

### 2.6 Genomic differences between the M2 mutant and the WT strain

Both the wild type (WT) and M2 mutant strains were sequenced to approximately 600x coverage using Illumina NovaSeq with 150bp paired-end reads. The sequences were mapped to the reference strain *Novacetimonas hansenii* (former name *Gluconacetobacter hansenii*) ATCC 53582 (NCBI-GenBank: GCA_900016225.1). Mapped reads were analyzed for the presence of single nucleotide polymorphisms (SNPs) as well as insertions or deletions (indels).

To identify mutations potentially associated with the phenotype observed in the M2 mutant, a comprehensive list of differences to the reference strain that are present in both the WT and the M2 strain was first compiled. Subsequently, all these changes found in both strains were eliminated, retaining only those mutations/insertions exclusive to the M2 strain. Missense variants and frameshift mutations with a moderate to high predicted impact on protein sequence (and potentially function) were specifically selected. These unique mutations/insertions in M2 are hypothesized to contribute to the mutant phenotype. The mutations specific to the M2 mutant are summarized in Table 1.

**Table 1:** **Mutation identified as exclusive in M2 strain.** Unique mutations found in M2 mutant when compared to ancestor WT strain.

Position (type of genetic mutation)^a^	Gene product	Function	Codon change	Amino acid change
1034551 (frameshift_variant)	AMP-binding protein	Metabolism	c.725_726ins	p.Trp243fs
128054 (missense_variant)	Cox15/ctaA family protein	Energy production	c.319G > A	p.Glu107Lys
755292 (missense_variant)	Bifunctional (P)ppGpp synthetase/guanosine-3′,5′-bis (Diphosphate) 3′-pyrophosphohydrolase	Adaptation (stringent response)	c.2071C > T	p.Arg691Cys
806488 (missense_variant)	Methyltransferase domain-containing protein	Regulation	c.275C > T	p.Ser92Leu
1518481 (frameshift_variant)	Methyltransferase RsmI	Regulation	c.403delC	p.His135fs
2188627 (missense_variant)	SCO family protein	Energy production	c.406C > T	p.Arg136Cys
1889848 (missense_variant)	RpoD	Regulation	c.934A > G	p.Lys312Glu
3151567 (missense_variant)	Hint domain-containing protein	Unknown	c.377C > T	p.Ala126Val

a Mutations listed are exclusive to the M2 mutant strain, i.e., not present in the WT strain. Missense variants and frameshift mutations with a moderate to high predicted impact on protein sequence and function were specifically selected. These mutations are hypothesized to contribute to the increased bacterial cellulose production phenotype observed in the M2 strain.

Mutations across various functional groups in mutant M2 were identified. In the metabolism category, a mutation was found in a gene encoding AMP-binding protein. Among adaptation systems, a mutation was identified in the bifunctional (p)ppGpp synthetase/guanosine-3′,5′-bis(Diphosphate) 3′-pyrophosphohydrolase (RelA/SpoT), which should affect the alarmone or second messenger (p)ppGpp. Two mutations potentially affecting energy metabolism were observed in the Cox15/CtaA family protein and the SCO family protein. Three mutations were found in regulatory factors, i.e., the vegetative sigma subunit of RNA polymerase (RpoD), the methyltransferase RsmI, and another uncharacterized methyltransferase domain-containing protein. Finally, one mutation was found in a gene encoding a Hint domain-containing protein of unknown function. Which of these mutation(s) is or are causally related to enhanced BC production and the observed changes in BC properties, will be discussed below and is a subject of future studies.

### 2.7 Untargeted exometabolomic profiling of culture media

**Fig. 6:**
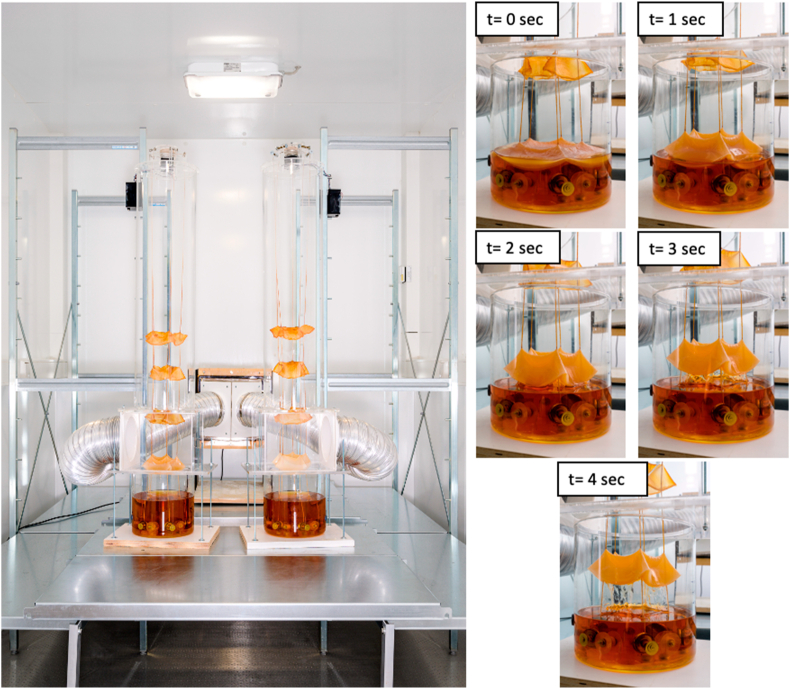
**Automated elevator system for harvesting BC.** This experimental setup was employed for growth of *G. hansenii* within a controlled semi-sterile chamber equipped with an automated elevator system. The automated elevator periodically lifted BC every 7 days using an array of vertical textile threads connected to the elevator (right images from t = 0sec to t = 4sec). Sampling outlets were used to extract culture media at different time points, enabling an in-depth exploration of metabolite alterations in the culture media.

Exometabolomics, also referred to as metabolic foot printing, is a specialized field within metabolomics that primarily investigates alterations in extracellular metabolites. In this study, untargeted exometabolomic profiling were conducted to examine metabolite uptake and secretion associated with both the WT and mutant M2 strains. To improve reproducibility, ensure continuity throughout the harvesting experiment, and minimize contamination risks, a novel experimental setup was employed [20]. This system involved the growth of *G. hansenii* within a controlled semi-sterile chamber equipped with an automated elevator system (Fig. 6). The elevator periodically lifted the bacterial cellulose (BC) using an array of vertical textile threads connected to the elevator (every 7 days). Sampling outlets were strategically integrated into the container to extract culture media at various time points, enabling an in-depth exploration of metabolite alterations in the culture media. This approach provided a comprehensive understanding of metabolite uptake and release dynamics by *G. hansenii* during cultivation. Finally, a comparative analysis of the exometabolomes was conducted for the M2 mutant and WT strains.

Glucose and gluconic acid were initially selected as metabolic reference biomarkers (Table 2) and compared their concentrations in the culture media of WT and M2 at day 0 and day 28. At day 28, glucose levels in both WT and M2 culture media were reduced but did not differ significantly (Table 2). Similarly, no significant difference in gluconic acid levels was observed between WT and M2. These findings suggest that both WT and the M2 mutant exhibit similar rates of glucose consumption and gluconic acid release after 28 days, with no notable differences in the metabolic processing of these compounds over the study period.

**Table 2:** **Fold change in metabolite features.** Glucose and d-gluconic acid levels in WT and M2 culture media at day 7 and day 28. Fold change represents magnitude of change of metabolites between the two groups (culture media). The statistical significance *p*-value of the ratio fold-change for each metabolite was calculated based on a *t*-test (significance p-value <0.005).

Culture media	Metabolite	Fold change	Log_2_ Fold change	Adjusted *p* value
**WT: day 0 versus day 28**	Glucose	2,6332^a^	1396	0,00072
	d-gluconic acid	0,0024^b^	−8702	0,00027
**M2: day 0 versus day 28**	Glucose	2,565^a^	1359	0,00433
	d-gluconic acid	0,0028^b^	−8480	0,00002

a Consumption of glucose and lower level at day 28 compared to day 0.

b Increase level of d-gluconic acid at day 28 compared to day 0.

**Fig. 7:**
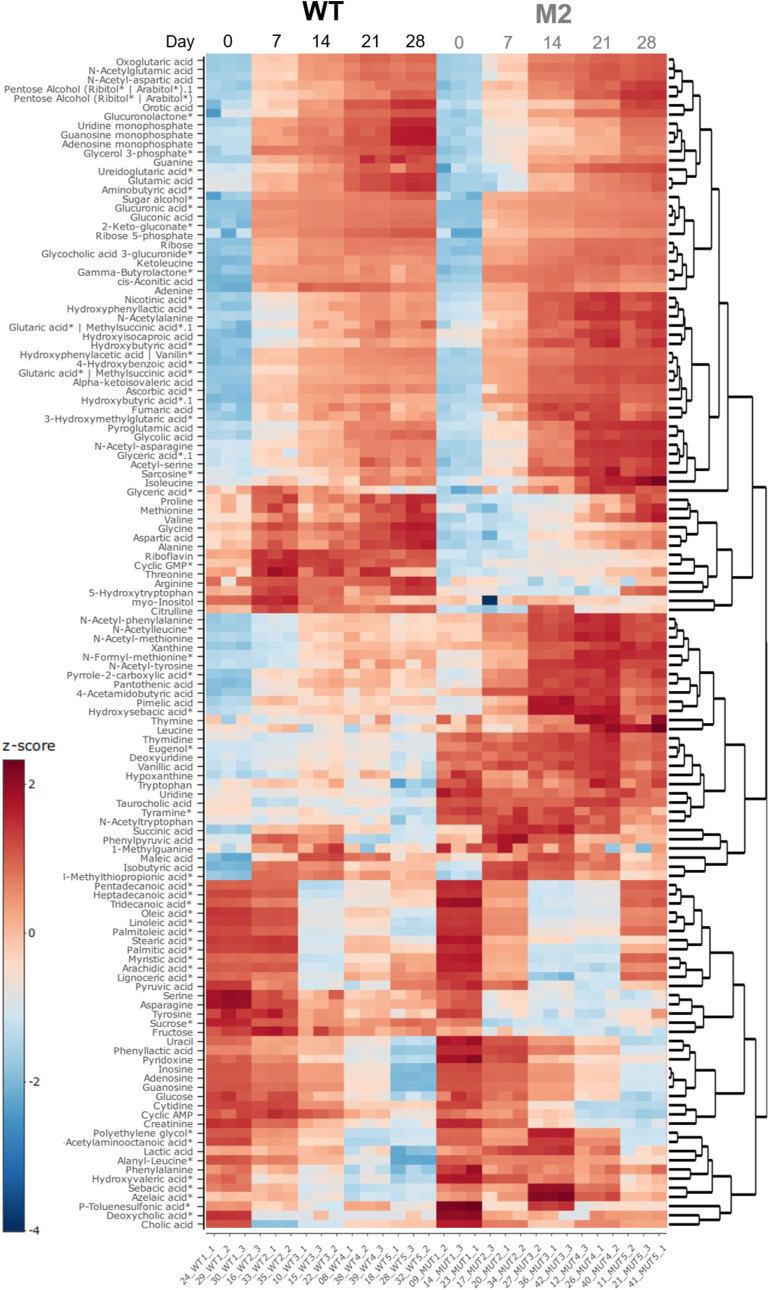
**Heatmap representing the footprint of WT and M2 mutant exometabolomes during 28 days of growth.** The heatmap shows the variation in metabolites week by week during the harvesting experiment. Metabolite levels are z-score transformed, with blue indicating lower-than-average levels and red indicating higher-than-average levels. Dendrograms on the side represent hierarchical clustering of metabolites, where branch length reflects their degree of similarity. Metabolite features shown are Level 1 confidence identifications, with asterisks (∗) indicating Level 2 annotations. (For interpretation of the references to colour in this figure legend, the reader is referred to the Web version of this article.)

However, the exometabolome analysis of WT and M2 revealed significant differences in the level of various other metabolites (Fig. 7). Notably, several metabolites were found in higher proportions in the culture media of the M2 mutant at day 28, including linoleic acid (polyunsaturated fatty acid, C18:2), oleic acid (monosaturated fatty acid C18:1), palmitoleic acid (monosaturated fatty acid C16:1), heptadecanoic acid (saturated fatty acid, C17:0), pentadecanoic acid (saturated fatty acid, C15:0), tridecanoic acid (saturated fatty acid, C13:0).

Intriguingly, nucleotides such as adenosine 3′-monophosphate (AMP), adenosine 3′5′-cyclic monophosphate (cAMP), guanosine 5′-monophosphate (GMP) and guanosine cyclic monophosphate (cGMP) were found in lower abundance in the exometabolome of the M2 mutant at day 28 (Fig. 7 and Fig. S7). The untargeted metabolomic profiling of metabolites from both WT and M2 mutant strains show a clear alteration in metabolic pathways, which may provide valuable insights into the metabolic dynamics of the M2 strain.

## 3 Discussion

Bacterial cellulose is a promising biomaterial. Its purity, biocompatibility, high water holding capacity and enhanced mechanical properties make it very attractive to several sectors including biomedicine, pharmaceutics, and cosmetics [8,24,25]. Currently, the main obstacles preventing large scale commercial application are high cost and low-yield production. Thus, it is urgent to develop new strategies to increase the productivity and yield of BC. In this study, a new approach that combines static culturing and intermittent harvesting was explored, which allows not only to enhance the BC production, but also improve cellulose structural and mechanical properties. Quite remarkably, the simple intermittent harvesting strategy not only had strong effects on BC properties but also led to the emergence of a mutant with higher BC-yields and distinct BC architecture.

In static growth, cellulose becomes a barrier for the nutrient transfer to the active cells at the top layer in the later stages of incubation [26]. In addition, Krystynowicz et al. [22] showed that forming layers of cellulose by *Acetobacter xylinum*, which is closely related to *G. hansenii*, limits the access to oxygen for the cells beneath the pellicle [22]. As a strictly aerobic bacterium, *G. hansenii* then starves for oxygen and cannot perform aerobic respiration efficiently limiting its growth [27]. Here the number of living and culturable cells was confirmed to decrease as the pellicle got thicker over time (Fig. S1). Accumulation of toxic metabolites and acidification of the medium affect planktonic cells physiology and BC productivity[14,18,26]. Previous studies showed that the lower the cell density, the lower the productivity of bacteria will be [28,29]. To overcome these limitations of static cultivation, alternative methods were explored [15,18,19,30]. Wu and Li [19] demonstrated that using a rotating disk bioreactor improved oxygen transfer and nutrient distribution, leading to enhanced bacterial cellulose yield and mechanical properties [19]. Similarly, Hornung et al. [18] optimized BC production using a stirred-tank bioreactor, where controlled agitation and aeration significantly increased cellulose formation [18].

With this work, the whole cellulose pellicle is proposed to be harvested every week, thereby removing the “active” cellulose producer cells along with the BC. Surprisingly, a new pellicle of cellulose was rapidly formed and visible after 2–3 days, which suggests that a remaining subpopulation of planktonic *G. hansenii* in the medium 1) migrated to the air-liquid interface, 2) became “active” cellulose producer cells and 3) formed a new BC pellicle. Sequentially harvesting the BC pellicles not only improved the production of cellulose, but also increased or prolonged glucose consumption and bacterial viability (Fig. 1).

Factors such as the cultivation process, time as well as composition of media are known to affect cellulose properties from the macroscopic to the nanoscopic levels, the different length scales being closely related through hierarchical organization [31]. Indeed, cellulose crystallinity has been suggested to affect the mechanical stiffness of individual cellulose ribbons [32]. Also, the relationship between the crystallinity of a cellulosic material and its stiffness was previously established [33] and can explain the correlation between the E-moduli and the crystalline index of the dried pellicle, which both increase until the 11th harvest (Fig. 2). Even if CI absolute values should be taken with caution, note that the CI values determined on the oven-dried pellicles are within the range of values reported for BC produced by *G. hansenii* [34]. How exactly bacteria can crystallize cellulose is not fully understood, but it has been shown that crystallization is a cell directed process and that the rate of polymerization and the rate of crystallization are coupled [35]. A change in cellulose CI could thus reflect an adaptation to the culture conditions. This hypothesis is supported by the CI difference measured between the two sides of the pellicle (medium side and air side), which undergoes a clear change throughout the intermittent harvesting experiments (Fig. 2d and e). These results suggest the existence of a gradient or variations of the crystallinity of the cellulose across the biofilm. As biofilm formation mostly happens at the air side and previously formed cellulose layers move downwards into the medium [36], it could be argued that these changes reflect the time course of biofilm formation. However, other factors affecting cellulose crystallinity as the different pellicle “ages” or “matures”, cannot be excluded. Interestingly, the increasing crystallinity observed in biofilms during the first two cycles of harvesting correlated with a decrease in BC crystallite size (Fig. 2f). While a direct correlation between crystallite size and CI might be expected, these changes could also be interpreted as a possible adaptation mechanism for more efficient resource use in case of shortage. Smaller crystallites might result from fewer glucan chains within a microfibril, corresponding to decreased energy investment of bacterial cells. The effect of reduced ribbon stiffness from smaller crystallites could then be offset by higher crystallinity: smaller crystals but more of them (Fig. 2d). Bacteria with such traits are likely to outcompete others due to the reduced energy investment and glucose use [32]. After the 11th harvest however, the decrease in BC crystallinity (CI) and subsequent stiffness (E-modulus) (Fig. 2b and c) suggest that additional factors interfere with assembly and lead to more amorphous cellulose, while bacteria produce pellicles of similar mass as in the previous cycle (Fig. 1b and f).

Variants of *G. hansenii* producing cellulose with adapted characteristics that help them to grow better in nutrient-limited environment, such as mutant M2, were thus expected to emerge among the bacteria throughout the multiple harvesting experiments (Fig. 3a). Yet not all the BC property changes observed after several harvests in the original experiment, are found in the first harvest of BC produced by M2. Indeed, while the quantity of BC obtained with strain M2 is clearly higher compared to the WT, their resistance to deformation (E-modulus and maximum stress) is weaker, which is consistent with their smaller crystallinity index (Fig. 4, Fig. 5) [33]. However, the CI differences between the two sides of the oven dried samples, indicate that WT BC is more crystalline on the medium side whereas M2 BC is slightly more crystalline on the air side (although the difference did not appear significant) (Fig. 4e and f). This switch of crystallinity distribution reminds the progressive changes observed along the harvesting experiment (Fig. 2e). Further microscopy investigations of the cross-section of lyophilized BC pellicles also revealed mesoscopic differences between WT and M2 samples and between their respective air and medium sides (Fig. 5a). These observations suggest that the strategy acquired by *G. hansenii* upon nutrient shortage during the harvesting experiment, became encoded in the genetics of a sub-population from which M2 was isolated. Evolved bacteria are thus expected to apply this strategy leading to more crystalline BC earlier during the growth period of 7 days, and thus higher CI on the air side. Such a mutant that exhibits differences in the matrix architecture from the nano-to the macroscopic scales, is a great opportunity to further investigate and understand the processes underlying bacterial cellulose production and organization.

To understand the genetic basis for these observations, a genomic comparison of the WT and M2 mutant bacteria was conducted (Table 1). Several mutations were identified in genes encoding regulatory proteins that can significantly influence *G. hansenii* adaptation through various mechanisms. Notably, a significant missense mutation (resulting in an arginine to cysteine exchange) was identified in the gene encoding the bifunctional (p)ppGpp synthetase/guanosine-3′,5′-bis(diphosphate) 3′-pyrophosphohydrolase (RelA/SpoT). This enzyme, with both synthetase and hydrolase domains for making and degrading, respectively, the second messenger or alarmone (p)ppGpp, controls the stringent response. In response to environmental stress, SpoT and RelA synthesize the stringent response alarmones ppGpp and pppGpp by catalyzing the transfer of phosphate groups from ATP to GDP or GTP, respectively, generating AMP and GMP or GDP as byproducts [37]. Consistently, levels of AMP and GMP were higher with the WT strain than with the M2 mutant, where this stress response is likely to be altered due to the mutation in bifunctional enzymes.

This rather general stress response leads to an active reduction of molecular growth functions in response to limiting carbon/energy sources and/or general stress, which allocates remaining resources more towards survival-enhancing stress responses. The mutation present in strain M2 affects the C-terminal regions of this RelA/SpoT enzyme, which is involved in controlling the synthase/hydrolase switch and may well tune down the stringent response, which is compatible with a dysregulated metabolic response and significant changes in the exometabolomic content of amino acids and fatty acids [38,39]. Tuning down the stringent response would also explain continued growth and thus more cellulose production under conditions of increasing oxygen/energy limitation as observed here with our mutant M2. Similar mutations that shift the stress responses *vs* growth balance in favor of the latter have previously been observed with *Escherichia coli* under nutrient-limiting, but otherwise constant, i.e., non-further challenging conditions [40].

A frameshift mutation was identified in a gene encoding an AMP-binding protein, which shares 84,48 % homology with acetyl-CoA synthetase (ACS) gene found in *Komagataeibacter medellinensis* (Blast search, NCBI). It is important to note that ACS is not the primary enzyme responsible for generating acetyl-CoA during active growth. Instead, it plays a specialized role under specific conditions, such as the transition to stationary phase, when preferred carbon source like glucose are depleted. The mutation in the ACS gene likely compromises this pathway, reducing the ability to re-assimilate acetate under nutrient-limiting conditions. However, instead of exhibiting a marked metabolic shift to accommodate this deficiency, the M2 mutant appears to maintain a growth-focused strategy even as resources diminish. This delayed entry into stationary-phase physiology is indicative of a weakened or incomplete stringent response, which would typically suppress processes like energy-intensive fatty acid synthesis and prioritize survival-oriented adaptations. Exometabolomic profiling further supports this interpretation. The M2 mutant culture supernatant showed an accumulation of fatty acids at day 28 (Fig. 7). These elevated levels of extracellular fatty acids suggest continued synthesis despite resource scarcity, likely due to a dysregulated metabolic response. Under normal conditions, fatty acid production would be downregulated during nutrient limitation to conserve resources, but the M2 mutant appears to bypass this regulation. This metabolic dysregulation may have broader implications for the cell's physiology. Changes in fatty acid profiles can influence membrane properties, including fluidity, which may affect processes like bacterial cellulose (BC) secretion. Indeed, differences in BC nano- and micro-structures between the wild type (WT) and M2 mutant (Fig. 4, Fig. 5) may reflect these changes. A study on *K. hansenii* HDM1-3, for example, have shown that higher proportions of unsaturated fatty acids enhance membrane fluidity and support survival under acidic conditions [41].

In a recent study, Laurent et al. use UV-C mutagenesis and high-throughput screening for rapid identification of cellulose overproducers [30]. *Komagataeibacter sucrofermentans* cellulose overproducer strains were subsequently compared to native and control strains. Genomic analysis revealed that enhanced cellulose production in the evolved strains was associated with a 12-base pair deletion in the *clpA* gene, altering a critical binding domain of the ClpAPS protease complex. In contrast, the approach presented in this study relies on prolonged growth to drive natural mutation, mimicking evolutionary pressures for more ecologically relevant adaptations. This approach likely led to different mutations, particularly those linked to the stringent response, as it emphasizes long-term survival and adaptation. A similar prolongation of vegetative growth at the expense of stress survival mechanisms has also been found in *E. coli*, where long-term incubation under conditions of low nutrient availability but otherwise constant, i.e., non-stressing conditions, leads to the appearance of mutations that also down-tune the interlinked stringent and RpoS-dependent stationary phase responses [40,42].

The present results provide a starting point for development of grown cellulosic materials with tunable micro-, meso and macroscopic properties such as crystallinity, micro-structure, and stiffness. Having the ability to naturally manipulate the mechanical properties of BC can open new strategies to make BC attractive as a sustainable material for the future [43]. Further systematic investigations are required to better understand the role of various factors and how they determine BC properties. In such context, the BC elevator system appears as an ideal tool both for implementing the multiple harvest strategy to larger BC production and for understanding the mechanisms underlying its success. Ultimately, integrating the elevator design, the M2 variant, and recent approaches that enhance aligned bacterial cellulose production [44], could open new avenues for the biofabrication of cellulose-based materials with tunable anisotropic properties.

## 4 Material and methods

### 4.1 Bacterial strain and growth media

The bacterial strain used in this work was *Gluconacetobacter hansenii* ATCC® 53582, was maintained in glycerol stocks at −80 °C. The strain was cultured in Hestrin and Schramm (HS) medium which contained bacto-peptone 5 g/L, yeast extract 5 g/L, sodium phosphate anhydrous 2.7 g/L, and citric acid monohydrate 1.5 g/L [45]. Glucose (20 g/L) was sterilized separately by autoclaving, and aseptically added to the autoclaved medium for complete HS medium, allowing us to adjust its concentration flexibly when needed. The final concentration of glucose in HS medium was 2 %. To make solid media, 15 g/L of agar was added to the HS liquid medium. Culture media were stored at 4 °C and used within two weeks.

### 4.2 Cultivation and production of bacterial cellulose (BC)

*Static condition.* The pre-culture of *G. hansenii* was performed on HS agar plates. After 48h–72h of growth, colonies were collected by taking five loops of the culture, which were resuspended in 5 mL of HS medium. The 5 mL were vortexed for 3 min and subsequently added to each of the six 600 mL glass beakers filled with 400 mL of HS media to achieve an inoculum concentration of approximatively 10^8^ CFU/mL. The cultures were then cultivated for 7, 14, 21, 28 and 35 days under static condition. Six 600 mL glass beakers filled with 400 mL of HS media were used for this study (N = 6). A sample of medium was taken on day 0 for analytical methods (control). Afterwards samples of medium were taken on day 7, 14, 21, 28 and 35. Experiments were repeated twice using 5 duplicates each time.

*Harvest method.* To optimize cellulose production, intermittent harvests were applied to the static growth condition described above. This method was called the “harvest method”. As described for the static condition, a preculture was prepared and used to inoculate a glass beaker filled with 400 mL of HS. Cultures were kept at 28 °C for 7 days, and the cellulose pellicle was then manually harvested using sterile tweezers and scalers. Afterwards, the beakers were placed back at 28 °C for another 7 days. The harvesting process was repeated every 7 days until day 35. One mL of culture media was taken on day 0 and then every 7 days for analytical analysis. The harvest of BC induced a decrease of media volume. To further extend the growth of *G. hansenii* and BC production, the culture was thus refilled with fresh media at day 35 to reach 400 mL and placed back the beakers at 28 °C. Similarly, BC were harvested, and samples were taken every 7 days for another 4 weeks, and fresh media was added again to extend the experiment up to 92 days.

### 4.3 Purification of BC and analysis of BC yield

In static conditions, separate cultures were simultaneously grown, and at each time point, a culture was taken apart and the BC pellicle were collected. For the harvest method, the same culture is used and a BC pellicle is harvested weekly. Harvested cellulose pellicles were either directly processed (fresh BC), or oven dried (oven dried BC) or frozen at −80 °C after freeze-drying. Fresh BC pellicles were collected, washed thoroughly with distilled water to remove medium components, and treated with NaOH 0.1 M at 60 °C for 1 h to remove bacterial cells [46]. Later, BC was washed again 3 times with deionized water at 60 °C (30 min) and then cooled at room temperature. BC were weighed and then freeze dried until a constant weight was reached to evaluate the BC yield in g/L. BC production is then reported as the mass of wet (before) or dry (after) cellulose per liter of the medium (g/L).

### 4.4 Isolation and characterization of mutants

Medium from 92-day old culture was plated on HS agar medium at 30 °C for 3–4 days. Individual colonies were picked and spread again on HS agar plates. Single colonies were picked and grown in 5 mL of HS medium under static condition and 30 °C for 4 days. Strain that showed thicker cellulose pellicles were picked and tested again in 600 mL glass beakers filled with 400 mL of HS media and compared to WT strain grown in the same conditions. Pellicles were collected as described above and weighed to be compared to the WT BC layers. A mutant called mutant M2 with enhanced cellulose mass was isolated for this study. BC production of WT strain and mutant M2 was reported as the mass of wet or dry cellulose per liter of the medium (g/L) and in percentage of increased production. The thickness of wet BC from WT and M2 mutant pellicles was measured manually.

### 4.5 Analytic methods

a) Bacterial growth

To determine bacterial growth of cells in media (planktonic cells), colony-forming units (CFU) were determined using serial dilution method from samples taken at different time points [47]. The number of viable and culturable bacteria in the media was counted after 5 days of incubation (CFU/mL). The ratio of cellulose-producing cells or Cel^+^ (rugous and star-shaped colony) to cellulose non-producing cells or Cel^−^ (smooth and round-shaped colony) was also noted and compared over incubation time (Fig. S2).

b) Glucose and gluconic acid measurement

From medium samples (1 mL) collected from each culture and at different time points. d-glucose and d-gluconic acid were measured by enzymatic kits (Megazyme Ltd., Bray, Ireland) according to the manufacturer's instructions. Measurement was repeated twice using triplicates.

c) pH measurement

pH was measured with a pH-meter SI-Analytics Lab 850 BlueLine 14 (Mainz, Germany) and an automatic titrator Mettler Toledo FiveEasyTM, equipped with a InLab micro electrode (Grieβen, Germany).

### 4.6 Characterization of BC pellicles

a) Tensile tests

Tensile tests were performed on the BC samples to evaluate their mechanical properties. Oven dried BC samples were cut using scissors to obtain strips of dimensions 8 mm x approximatively 8 cm. The thickness of the strips was measured at three points using a digital micrometer screw and the sample cross section area (A) was calculated by assuming a rectangular cross section. BC strips were subjected to tensile measurement using a Zwick LF7M10 universal testing machine equipped with a 10-kN load cell. The distance between the clamps was set to 25 mm (L_0_) and the samples were tested with an extension rate of 2 mm/min. A pre-force of 0,5 N was applied before starting the measurement to remove potential buckling induced during sample positioning. At least 3 specimens were measured per group. The tensile stress σ was then obtained by normalizing the force F applied on the sample, by the sample cross section area A. The axial strain *ε* was calculated from the elongation length (ΔL) normalized by L_0_. The stiffness of the sample E (also called E-modulus) represents the resistance to deformation (E = σ/*ε*) and was estimated from the initial slope of each stress-strain curve by applying a linear fit between 0 and 0.5 % strain, i.e., in the linear elastic region.

b) X-Ray Diffraction (XRD)

XRD analysis was used to investigate the crystallinity of the BC. Approximately 2 × 2 cm pieces were cut from oven dried BC with scissors. Samples were positioned on the diffractometer XRD-D8 (Bruker, Karlsruhe) equipped with a monochromatic CuKα source emitting radiation at λ = 0.1542 nm. Diffraction spectra were acquired with angular steps of 0.02° and 1 s integration per step. Each sample was measured on both the air and medium sides. The XRD spectra were exported with the software DiffracSuite Eva version 4.3 (Bruker, Karlsruhe). The crystallinity index (CI) and the average crystallite dimensions were determined using custom made algorithms written in Python, following the procedure introduced by Ref. [33,33]). Briefly, analyses were restricted to the 2θ range between 10° and 30°, which contains the three peaks corresponding to the three main crystalline directions of cellulose at 2θ = 14.8° (1-10), 2θ = 16.5° (110) and 2θ = 22.2° (200). This data range was subjected to a two-point baseline correction and peak fitting with Gaussian functions was performed on both the sharp crystalline peaks and the broad amorphous bump using the Python library *lmfit* [33]. The CI was then determined as CI = A_cryst_/A_total_ following the peak area-based method, where A_cryst_ is the sum of the area of all crystalline peaks, and A_total_ is the combined area of crystalline and amorphous peaks. The crystallite dimensions were calculated using the Scherrer equation (1):

(1) $$\tau =\frac{K.\lambda }{\beta .\cos\;\theta }$$

where *τ* is the size in nm of the crystallite in the direction perpendicular to the crystalline direction considered, *K* is a constant of value 0.94, λ is the X-ray wavelength, *θ* is the angular value of the peak corresponding to the respective crystalline direction, *β* is the full width at half maximum of the same peak [48]. Note that because an instrumental peak broadening effect cannot be considered in this analysis, only the comparison between CI values or between crystallite dimensions will be considered in further interpretations (and not absolute values).

c) Wide angle X-Ray scattering (WAXS)

Wide angle X-rays scattering was performed on cross-sections of BC samples obtained from the WT and M2 mutants after 7 days of growth in fresh medium. About 2 mm thick cross-sections were cut with a blade out of partially thawed BC samples, and then oven dried between two weighing boats. The resulting sections were finally mounted in aluminum frames and hold between two pieces of Kapton film. Small-angle/wide-angle X-ray scattering (SAXS/WAXS) measurements were performed at the μSpot beamline at the BESSY II synchrotron (Helmholtz Zentrum für Materialien und Energie, Berlin, Germany). The complete diffractograms are shown in Fig. S5. The measurements were carried out using a B4C/Mo Multilayer (2 nm period) monochromator and an energy of 15 keV. A sequence of pinholes was used to select a 30 × 30 μm^2^ spot size. Data were normalized over the primary beam intensity and the background was subtracted. Transmission through the sample was calculated from an X-ray fluorescence (XRF) signal collected from a lead beam stop using a RAYSPEC Sirius SD-E65133-BE-INC detector. The XRF detector was equipped with an 8 μm beryllium window, where the primary beam intensity was monitored using an ion chamber. Scattering data were collected with a Dectris Eiger 9 M detector with 75 × 75 μm^2^ pixel area. On each sample, spots were measured every 150 μm to obtain profiles across the BC section, and diffraction patterns were collected with an exposure time of 15 s. Further data processing and reduction was done using the directly programmable data analysis kit (DPDAK). Diffraction patterns were radially integrated and the scattered intensity I(q) was calculated as a function of the momentum transfer q, defined in (2):

(2) $$q=\frac{4\pi }{\lambda }\sin\left(\frac{\theta }{2}\right)$$

with λ and θ the photon wavelength and the scattering angle, respectively. The sample-to-detector distance was set to approximately 330 mm (0.1< q < 30 nm^−1^) and calibrated by using quartz. Instrumental settings such as beam divergence, beam profile, sample to detector distance, detector pixels size and binning constant and energy resolution were used to calculate the instrumental broadening as a function of the momentum transfer. Decoupling of peak width and instrumental smearing was considered during peak fitting to properly estimate crystallite size. Data were analyzed and plotted respectively with in-house Python-based (Python 3.7) and Matlab-based (MATLAB R2021a) software.

d) Environmental scanning electron microscopy (ESEM)

ESEM was used to observe the morphology of the cellulose network. BC samples washed in 0.1 M NaOH for 2 h at 80 °C and then in double distilled water for another 2 h, were lyophilized (freeze dried) for 24 h at a pressure of 0.65 mbar and temperature of −25 °C, and finally cut by hand with sterile razor blades. Samples were then mounted on a sample holder with conductive carbon tape. Images were obtained with a Quanta 600F ESEM (FEI, Hillsboro, OR, USA) under low-vacuum condition and an accelerating voltage of 5.0 kV. To produce images spanning the entire cross sections of the BC, individual images were taken along the cross section with 20 % overlap between images.

### 4.7 Genome sequencing

DNA isolation, library preparation, whole genome sequencing and genome variant detection was performed by Eurofins Genomics (Ebersberg, Germany). Library was sequenced on an Illumina MiSeq (Illumina) using 150 bp, paired end reads to a coverage of approximately 600x. Before assembly, reads were quality controlled using FastQC. Mapping to the reference *G. hansenii* NBRC 3288 genome was done using BWA MEM and run through Sentieon framework. Assembled genome was then subjected to quality control using Quast. The analysis of the SNP used in the study was performed using ARIBA pipeline.

### 4.8 Exo-metabolic profiling: LC-MS/MS analysis

LC-MS grade water and acetonitrile were obtained from Th. Geyer (Germany). High-purity ammonium hydroxide and formic acid were purchased from Merck (Germany). For internal standards, a labelled standard mix (MSK-MET1-1; Cambridge Isotope Laboratories, MA, USA) was used.

a) Sample collection.

For metabolite analysis, samples of 500 μL were collected (in triplicate) and mixed with 1.5 mL of HPLC grade methanol (Sigma Aldrich, Germany). Samples were centrifuged and transferred to new containers (3 x 300 μL). Samples were then dried using a SpeedDry Vacuum Concentrator. 300 μL of each extracted sample were dried under a stream of N_2_ and reconstituted in 100 μL 85 % acetonitrile (+3 % internal standard mix). After thorough vortexing, samples were shaken for 10 min and centrifuged at 15,000*g* and 4 °C for 10 min with a 5415R microcentrifuge (Eppendorf, Germany). The supernatants were then transferred to analytical glass vials, and the LC-MS/MS analysis was initiated within 1 h after the completion of the sample preparation.

b) LC-MS/MS analysis.

Analysis was performed following an established method [49], on a Vanquish UHPLC system coupled to an Orbitrap Exploris 240 high-resolution mass spectrometer (Thermo Scientific, MA, USA) in negative ESI (electrospray ionization) mode. Chromatographic separation was carried out on an Atlantis Premier BEH Z-HILIC column (Waters, MA, USA; 2.1 mm × 100 mm, 1.7 μm) at a flow rate of 0.26 mL/min. The mobile phase consisted of water (mobile phase A) and 85 % acetonitrile (mobile phase B), which were modified with a total buffer concentration of 10 mM ammonium acetate. The aqueous portion of each mobile phase was adjusted to pH 9.0 by addition of ammonium hydroxide. The following gradient (20 min total run time including re-equilibration) was applied (time [min]/%B): 0/100, 2/100, 14/70, 14.5/60, 16.5/60, 16.8/100, 20/100. Column temperature was maintained at 40 °C, the autosampler was set to 4 °C and sample injection volume was 10 μL. Analytes were recorded via a full scan with a mass resolving power of 120,000 over a mass range from 70 to 800 *m/z* (scan time: 100 ms, RF lens: 70 %). To obtain MS/MS fragment spectra, data-dependent acquisition was carried out (resolving power: 15,000; scan time: 22 ms; stepped collision energies [%]: 30/50/70; cycle time: 900 ms). Ion source parameters were set to the following values: spray voltage: −3500 V, sheath gas: 30 psi, auxiliary gas: 5 psi, sweep gas: 0 psi, ion transfer tube temperature: 350 °C, vaporizer temperature: 300 °C.

All experimental samples were measured in a randomized manner. Pooled quality control (QC) samples were prepared by mixing equal aliquots from each processed sample. Multiple QCs were injected at the beginning of the analysis to equilibrate the analytical system. A QC sample was analyzed after every 5th experimental sample to monitor instrument performance throughout the sequence. For determination of background signals and subsequent background subtraction, an additional processed blank sample was recorded. Data was processed using MS-DIAL (50) and raw peak intensities were extracted for relative quantification. Raw data was normalized via total ion count of all detected analytes and via internal standards. Feature identification was based on accurate mass, isotope pattern, MS/MS fragment scoring (level 2 confidence identification) and for available standards also via retention time matching (level 1 confidence identification) [49,50].

### 4.9 Statistical analysis

Analysis of variance (ANOVA) tests and Tukey's multiple-comparison posttest were performed to compare cellulose productivity over time and between the two different growth conditions. Statistical analyses were performed with GraphPad Prism 9 (GraphPad Software, Inc., San Diego, CA, USA). For the E moduli and CI comparisons, Mann-Whitney tests were performed with OriginPro 2021b (OriginLab Corporation, Northampton, MA, USA) (p = 0.05).

## CRediT authorship contribution statement

**N. Rackov:** Writing – review & editing, Writing – original draft, Investigation, Formal analysis. **N. Janßen:** Writing – review & editing, Writing – original draft, Visualization, Software, Methodology, Investigation, Formal analysis. **A. Akkache:** Writing – review & editing, Formal analysis. **B. Drotleff:** Writing – review & editing, Visualization, Methodology, Investigation, Formal analysis. **B. Beyer:** Writing – review & editing, Methodology, Conceptualization. **E. Scoppola:** Writing – review & editing, Software, Methodology, Investigation. **N.E. Vrana:** Writing – review & editing. **R. Hengge:** Writing – review & editing, Resources. **C.M. Bidan:** Writing – review & editing, Writing – original draft, Visualization, Supervision, Resources, Project administration, Methodology, Formal analysis, Conceptualization. **S. Hathroubi:** Writing – review & editing, Writing – original draft, Visualization, Supervision, Project administration, Methodology, Investigation, Formal analysis, Conceptualization.

## Declaration of competing interest

The authors declare that they have no known competing financial interests or personal relationships that could have appeared to influence the work reported in this paper.

## Data Availability

Data will be made available on request.

## References

[1] Brooks R.E., Moore S.B. (2000). Alkaline hydrogen peroxide bleaching of cellulose. Cellulose.

[2] Amorim JDP de, Souza KC de, Duarte C.R., Duarte I. da S., Ribeiro F. de AS., Silva G.S. (2020). Plant and bacterial nanocellulose: production, properties and applications in medicine, food, cosmetics, electronics and engineering. A REVIEW. Environ Chem Lett.

[3] Choi S.M., Shin E.J. (2020). The nanofication and functionalization of bacterial cellulose and its applications. Nanomaterials.

[4] Ul-Islam M., Khan S., Ullah M.W., Park J.K. (2019). Comparative study of plant and bacterial cellulose pellicles regenerated from dissolved states. Int J Biol Macromol.

[5] Römling U., Galperin M.Y. (2015). Bacterial cellulose biosynthesis: diversity of operons, subunits, products, and functions. Trends Microbiol.

[6] Serra D.O., Hengge R. (2019). A c-di-GMP-Based switch controls local heterogeneity of extracellular matrix synthesis which is crucial for integrity and morphogenesis of Escherichia coli macrocolony biofilms. J Mol Biol.

[7] Florea M., Reeve B., Abbott J., Freemont P.S., Ellis T. (2016). Genome sequence and plasmid transformation of the model high-yield bacterial cellulose producer Gluconacetobacter hansenii ATCC 53582. Sci Rep.

[8] Zhong C. (2020). Industrial-scale production and applications of bacterial cellulose. Front Bioeng Biotechnol.

[9] Hur D.H., Rhee H.-S., Lee J.H., Shim W.Y., Kim T.Y., Lee S.Y. (2020). Enhanced production of cellulose in Komagataeibacter xylinus by preventing insertion of IS element into cellulose synthesis gene. Biochem Eng J.

[10] Park J.K., Hyun S.H., Jung J.Y. (2004). Conversion ofG. hansenii PJK into non-cellulose-producing mutants according to the culture condition. Biotechnol Bioproc Eng.

[11] Ryngajłło M., Jędrzejczak-Krzepkowska M., Kubiak K., Ludwicka K., Bielecki S. (2020). Towards control of cellulose biosynthesis by Komagataeibacter using systems-level and strain engineering strategies: current progress and perspectives. Appl Microbiol Biotechnol.

[12] Zhang H., Chen C., Yang J., Sun B., Lin J., Sun D. (2022). Effect of culture conditions on cellulose production by a komagataeibacter xylinus strain. Macromol Biosci.

[13] Souza SS de, Berti F.V., Oliveira KPV de, Cqp Pittella, Castro JV de, Pelissari C. (2019). Nanocellulose biosynthesis by Komagataeibacter hansenii in a defined minimal culture medium. Cellulose.

[14] Aswini K., Gopal N.O., Uthandi S. (2020). Optimized culture conditions for bacterial cellulose production by Acetobacter senegalensis MA1. BMC Biotechnol.

[15] Hsieh J.-T., Wang M.-J., Lai J.-T., Liu H.-S. (2016). A novel static cultivation of bacterial cellulose production by intermittent feeding strategy. J Taiwan Inst Chem Eng.

[16] Hornung M., Biener R., Schmauder H. (2009). Dynamic modelling of bacterial cellulose formation. Eng Life Sci.

[17] Zhong C., Li F., Liu M., Yang X.-N., Zhu H.-X., Jia Y.-Y. (2014). Revealing differences in metabolic flux distributions between a mutant strain and its parent strain gluconacetobacter xylinus CGMCC 2955. PLoS One.

[18] Hornung M., Ludwig M., Schmauder H.P. (2007). Optimizing the production of bacterial cellulose in surface culture: a novel aerosol bioreactor working on a fed batch principle (Part 3). Eng Life Sci.

[19] Wu S.-C., Li M.-H. (2015). Production of bacterial cellulose membranes in a modified airlift bioreactor by Gluconacetobacter xylinus. J Biosci Bioeng.

[20] Hathroubi S., Beyer B., Bertrand G., Favard M., Lartigaud D.O. (2021). MàJ Design, environnements techniques & pratiques exploratoires.

[21] Florea M., Hagemann H., Santosa G., Abbott J., Micklem C.N., Spencer-Milnes X. (2016). Engineering control of bacterial cellulose production using a genetic toolkit and a new cellulose-producing strain. Proc Natl Acad Sci USA.

[22] Krystynowicz A., Czaja W., Wiktorowska-Jezierska A., Gonçalves-Miśkiewicz M., Turkiewicz M., Bielecki S. (2002). Factors affecting the yield and properties of bacterial cellulose. J Ind Microbiol Biotechnol.

[23] Suryanto H., Muhajir M., Sutrisno T.A., Mudjiono Zakia N., Yanuhar U. (2019). The mechanical strength and morphology of bacterial cellulose films: the effect of NaOH concentration. IOP Conf Ser Mater Sci Eng.

[24] Amorim J., Costa A., Galdino C., Vinhas G., Santos E., Sarubbo L. (2019). Bacterial cellulose production using industrial fruit residues as subtract to industrial application. Chemical Engineering Transactions.

[25] Petersen N., Gatenholm P. (2011). Bacterial cellulose-based materials and medical devices: current state and perspectives. Appl Microbiol Biotechnol.

[26] Hornung M., Ludwig M., Gerrard A.M., Schmauder H.‐P. (2006). Optimizing the production of bacterial cellulose in surface culture: evaluation of substrate mass transfer influences on the bioreaction (Part 1). Eng Life Sci.

[27] Gomes R.J., Borges M. de F., Fortaleza C.E., Rosa M. de F., Castro-Gómez R.J.H., Spinosa W.A., Brazil ETA CEP 60511-110 (2018). Acetic acid bacteria in the food industry: systematics, characteristics and applications. Food Technol Biotechnol.

[28] Jung J.Y., Khan T., Park J.K., Chang H.N. (2007). Production of bacterial cellulose by Gluconacetobacter hansenii using a novel bioreactor equipped with a spin filter. Kor J Chem Eng.

[29] Shezad O., Khan S., Khan T., Park J.K. (2009). Production of bacterial cellulose in static conditions by a simple fed-batch cultivation strategy. Kor J Chem Eng.

[30] Laurent J.M., Jain A., Kan A., Steinacher M., Casimiro N.E., Stavrakis S. (2024). Directed evolution of material-producing microorganisms. Proc Natl Acad Sci USA.

[31] Wu Z., Chen S., Li J., Wang B., Jin M., Liang Q. (2023). Insights into hierarchical structure–property–application relationships of advanced bacterial cellulose materials. Adv Funct Mater.

[32] Ruan C., Zhu Y., Zhou X., Abidi N., Hu Y., Catchmark J.M. (2016). Effect of cellulose crystallinity on bacterial cellulose assembly. Cellulose.

[33] Poletto M., Júnior H., Zattera A. (2014). Native cellulose: structure, characterization and thermal properties. Materials.

[34] Fang L., Catchmark J.M. (2015). Characterization of cellulose and other exopolysaccharides produced from Gluconacetobacter strains. Carbohydr Polym.

[35] Benziman M., Haigler C.H., Brown R.M., White A.R., Cooper K.M. (1980). Cellulose biogenesis: polymerization and crystallization are coupled processes in Acetobacter xylinum. Proc Natl Acad Sci USA.

[36] Gromovykh T.I., Pigaleva M.A., Gallyamov M.O., Ivanenko I.P., Ozerova K.E., Kharitonova E.P. (2020). Structural organization of bacterial cellulose: the origin of anisotropy and layered structures. Carbohydr Polym.

[37] Chau N.Y.E., Ahmad S., Whitney J.C., Coombes B.K. (2021). Emerging and divergent roles of pyrophosphorylated nucleotides in bacterial physiology and pathogenesis. PLoS Pathog.

[38] Potrykus K., Cashel M. (2008). (p) ppGpp: still Magical?∗. Annu Rev Microbiol.

[39] Ronneau S., Hallez R. (2019). Make and break the alarmone: regulation of (p)ppGpp synthetase/hydrolase enzymes in bacteria. FEMS Microbiol Rev.

[40] Ferenci T. (2016). Trade-off mechanisms shaping the diversity of bacteria. Trends Microbiol.

[41] Li Y., Yan P., Lei Q., Li B., Sun Y., Li S. (2019). Metabolic adaptability shifts of cell membrane fatty acids of Komagataeibacter hansenii HDM1-3 improve acid stress resistance and survival in acidic environments. J Ind Microbiol Biotechnol.

[42] Ferenci T., Spira B. (2007). Variation in stress responses within a bacterial species and the indirect costs of stress resistance. Ann N Y Acad Sci.

[43] Li T., Chen C., Brozena A.H., Zhu J.Y., Xu L., Driemeier C. (2021). Developing fibrillated cellulose as a sustainable technological material. Nature.

[44] Murugarren N., Roig‐Sanchez S., Antón‐Sales I., Malandain N., Xu K., Solano E. (2022). Highly aligned bacterial nanocellulose films obtained during static biosynthesis in a reproducible and straightforward approach. Adv Sci.

[45] Schramm M., Hestrin S. (1954). Factors affecting production of cellulose at the air/liquid interface of a culture of acetobacter xylinum. Microbiology.

[46] Fei S., Fu M., Kang J., Luo J., Wang Y., Jia J. (2024). Enhancing bacterial cellulose production of Komagataeibacter nataicola through fermented coconut water by Saccharomyces cerevisiae: a metabonomics approach. Curr Res Food Sci.

[47] Liu K., Catchmark J.M. (2019). Enhanced mechanical properties of bacterial cellulose nanocomposites produced by co-culturing Gluconacetobacter hansenii and Escherichia coli under static conditions. Carbohydr Polym.

[48] French A.D., Cintrón M.S. (2013). Cellulose polymorphy, crystallite size, and the segal crystallinity index. Cellulose.

[49] Dekina S., Alexandrov T., Drotleff B. (2024). EMBL-MCF 2.0: an LC-MS/MS method and corresponding library for high-confidence targeted and untargeted metabolomics using low-adsorption HILIC chromatography. Metabolomics.

[50] Sumner L.W., Amberg A., Barrett D., Beale M.H., Beger R., Daykin C.A. (2007). Proposed minimum reporting standards for chemical analysis. Metabolomics.

