# Supplementary material for: Bacterial cellulose: Enhancing productivity and material properties through repeated harvest
Source: Biofilm. 2025 Mar 26;9:100276. doi: 10.1016/j.bioflm.2025.100276 (PMC11999647; doi:10.1016/j.bioflm.2025.100276)
Supplement: Multimedia component 1 [file mmc1.pdf]

<u>**Supplementary Information**</u>

Bacterial Cellulose: Enhancing Productivity and Material Properties Through Repeated Harvest

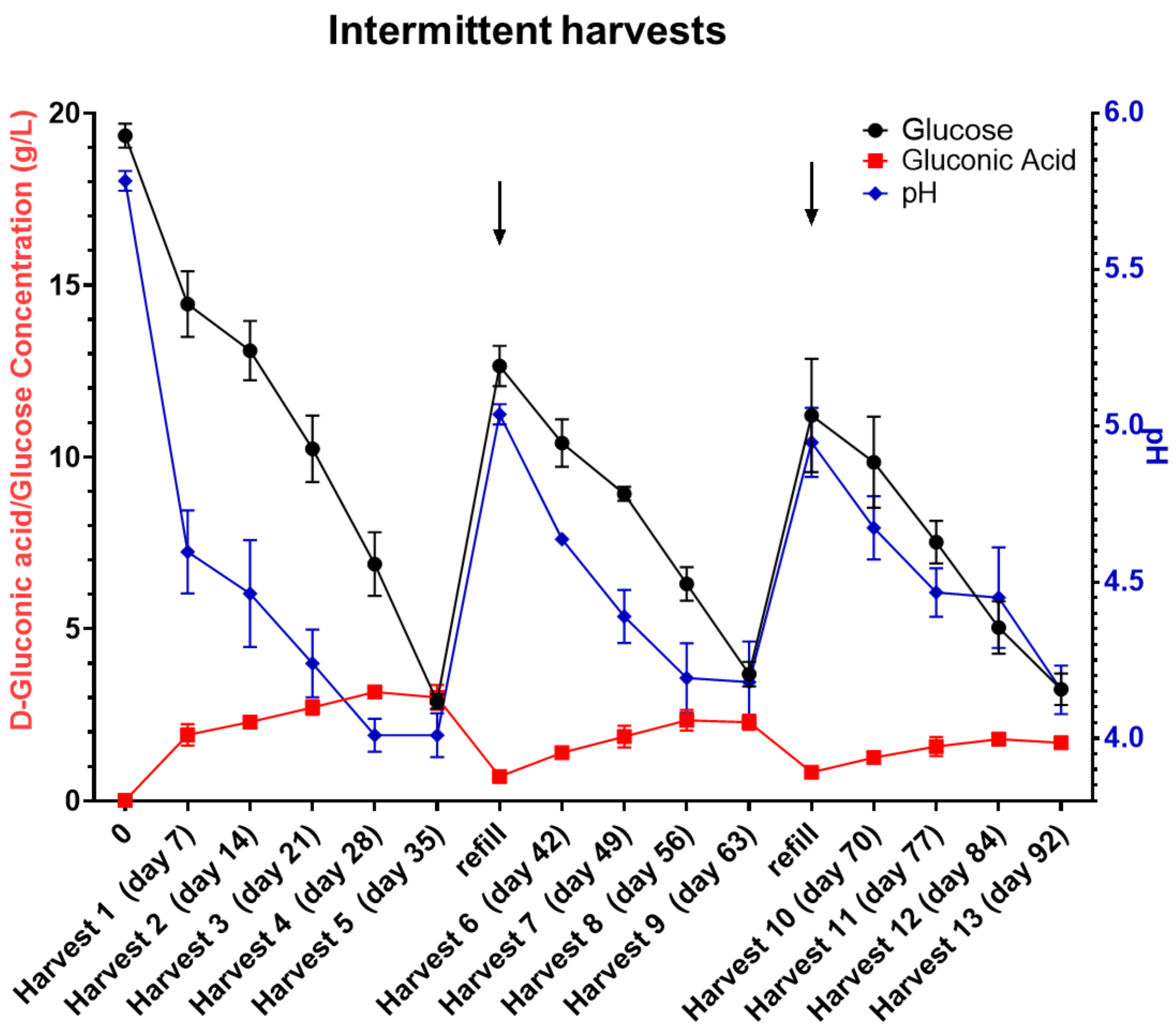

Fig. S1: Changes of pH, glucose and gluconic acid concentrations. Concentrations of glucose and gluconic acid, and pH measurement over time and with intermittent harvests.

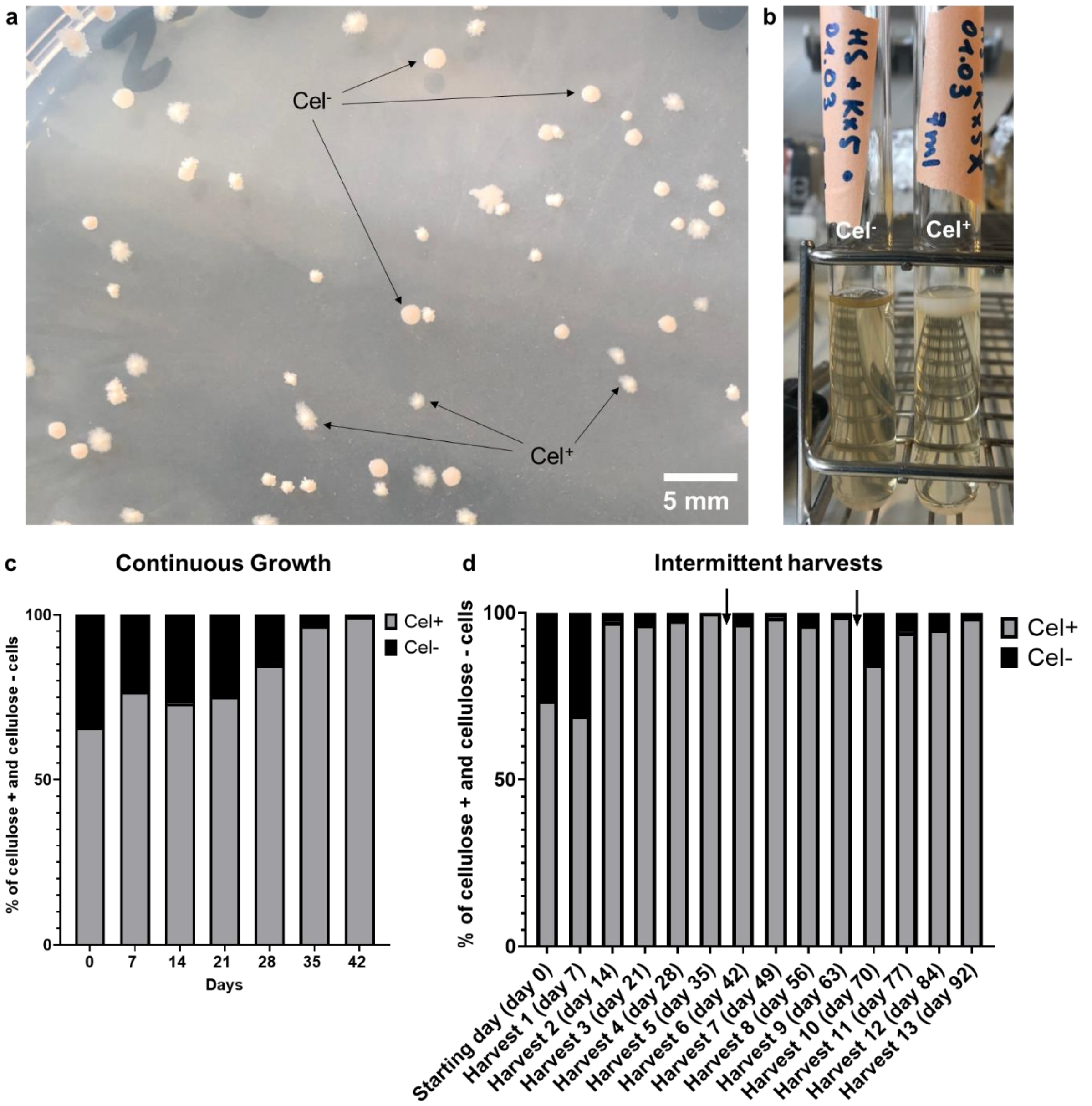

Fig. S2: Changes of colony physiology. (a) Colony morphology made by cellulose positive (Cel^+) and cellulose negative (Cel^-) *G. hansenii* bacteria on a HS agar substrate. (b) Results from their respective culture in liquid HS medium. (c-d) Percentage of cellulose positive (grey) and cellulose negative (black) obtained by CFU counting in the liquid medium during a culture with continuous growth (c) and intermittent harvests (d). Arrows represent the time where a replenish of the culture media was done.

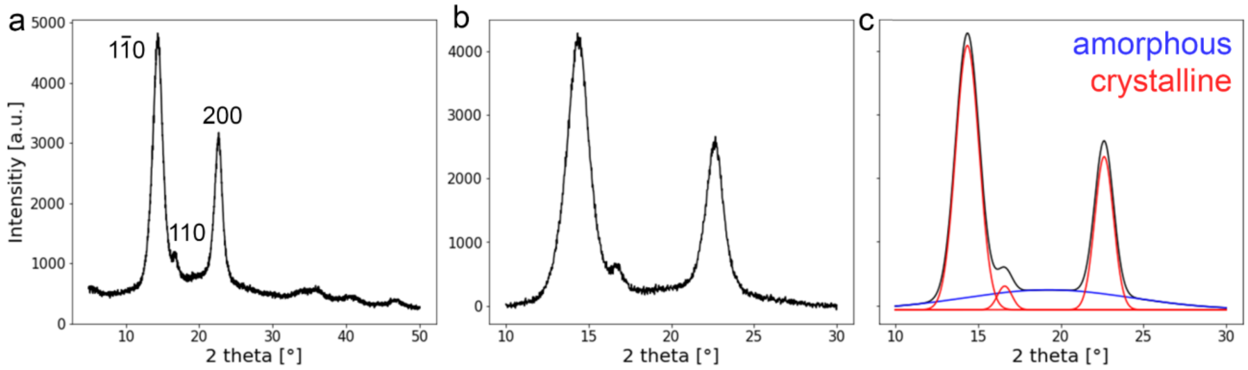

Fig. S3: XRD analysis. (a) Representative XRD diffractogram with crystalline peaks denoted by Miller indices. (b) Baseline corrected XRD diffractogram in the region 2θ = 10 to 30°. (c) Curve fitting of diffractogram and the corresponding gaussian components.

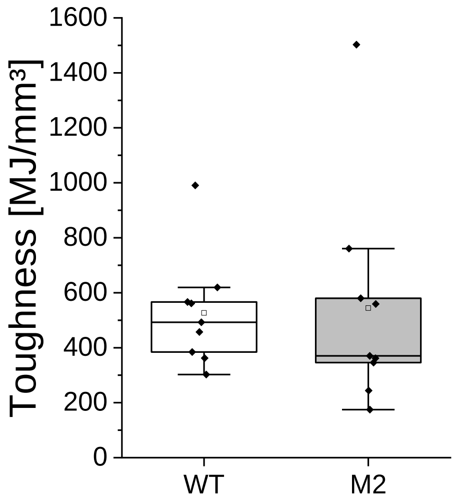

Fig. S4: Toughness of WT *vs* M2 BC pellicles, derived from the stress-strain curves displayed in Fig. 4a.

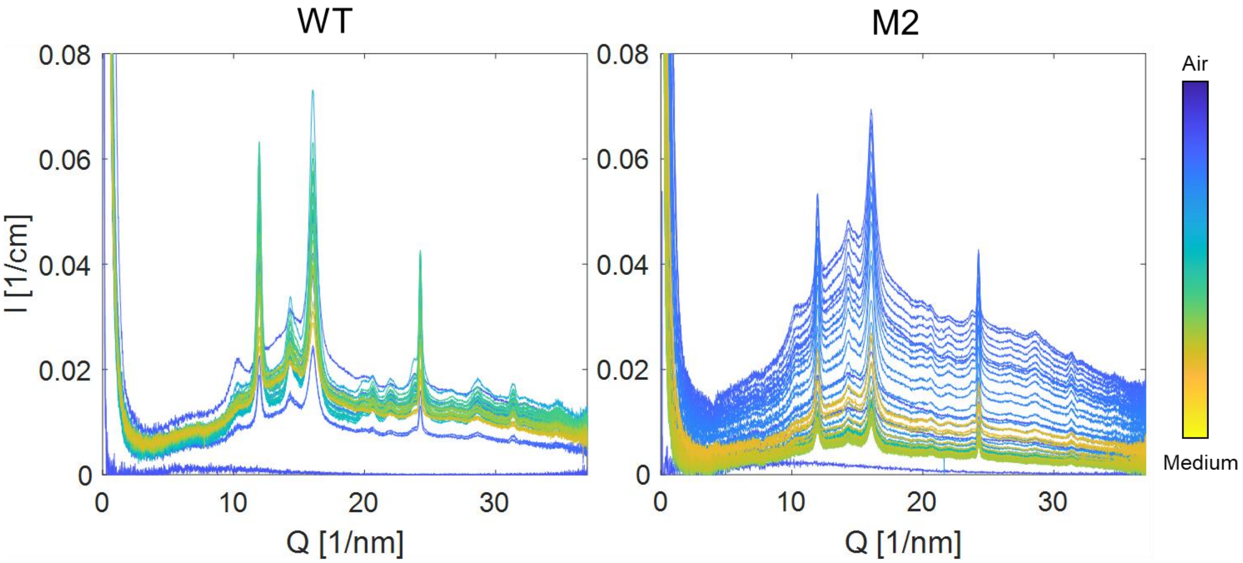

Fig. S5: Diffractograms obtained on cross-sections of BC pellicles from WT and M2 cultures using a synchrotron X-ray source. The corresponding crystallinity indices and crystal dimensions are presented in Fig. 5 b and c.

Table S1: Most prominent q values and corresponding 2θ values and d-spacing for the five main crystal lattice planes observed in the BC samples.

Lattice planes	(1-10)	(110)	(102)	(200)	(004)
2θ [°]	14.8	16.8	20.1	22.6	34.5
Q [nm^{-1}]	10.5	11.9	14.2	15.8	24.2
d-spacing [nm]	0.599	0.528	0.442	0.393	0.260

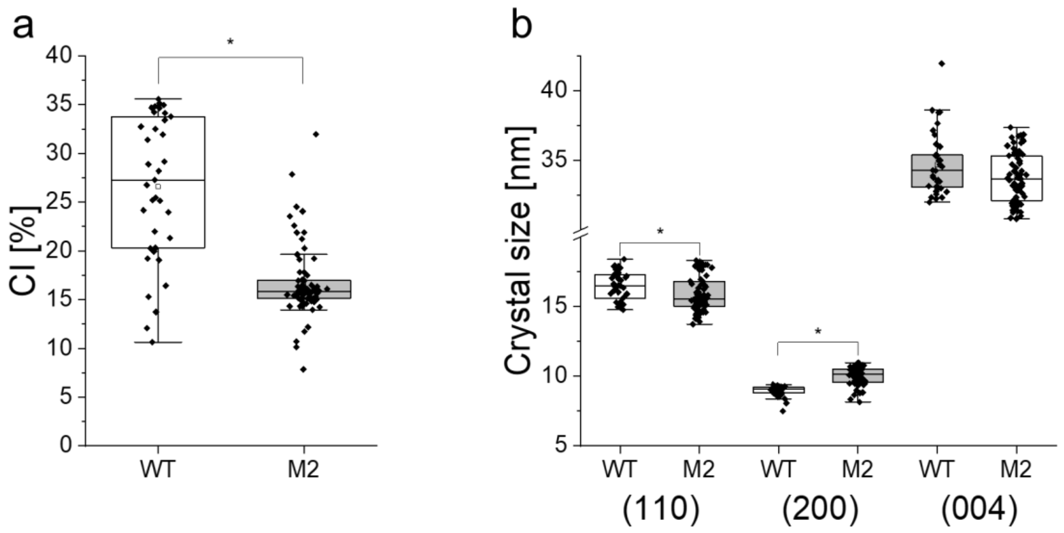

Fig. S6: (a) Crystallinity indices (CI) and (b) crystal dimensions of BC cellulose produced by WT vs M2 along their respective cross-section profiles, as derived from the diffractograms presented in Fig. S5.

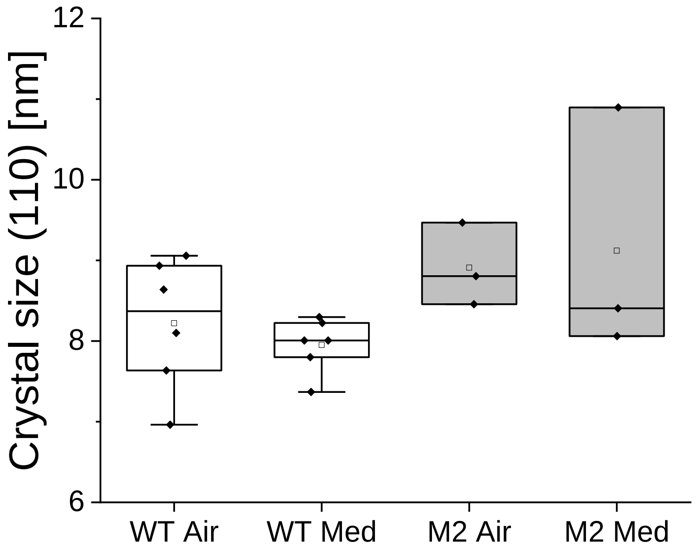

Fig. S7: BC crystal size determined from XRD measurements in the direction orthogonal to the cellulose lattice plane (110) on both the medium and the air sides.
